# Establishing Disorder-Specific and Transdiagnostic Neural Features of Psychiatric Disorders Through Large-Scale Functional Magnetic Resonance Imaging Meta-Analyses

**DOI:** 10.1192/j.eurpsy.2023.1156

**Published:** 2023-07-19

**Authors:** C. H. Miller, E. Pritchard, S. Saravia, M. Duran, S. L. Santos, J. P. Hamilton, D. W. Hedges, I. H. Gotlib, M. D. Sacchet

**Affiliations:** 1 Department of Psychology, California State University, Fresno, United States; 2 Department of Biomedical and Clinical Sciences, Linköping University, Linköping, Sweden; 3 Department of Psychology, Brigham Young University, Provo; 4 Department of Psychology, Stanford University, Stanford; 5 Department of Psychiatry, Harvard University, Cambridge, United States

## Abstract

**Introduction:**

Meta-analyses of functional magnetic resonance imaging (fMRI) studies have been used to elucidate the most reliable neural features associated with various psychiatric disorders. However, it has not been well-established whether each of these neural features is linked to a specific disorder or is transdiagnostic across multiple disorders and disorder categories, including mood, anxiety, and anxiety-related disorders.

**Objectives:**

This project aims to advance our understanding of the disorder-specific and transdiagnostic neural features associated with mood, anxiety, and anxiety-related disorders as well as to refine the methodology used to compare multiple disorders.

**Methods:**

We conducted an exhaustive PubMed literature search followed by double-screening, double-extraction, and cross-checking to identify all whole-brain, case-control fMRI activation studies of mood, anxiety, and anxiety-related disorders in order to construct a large-scale meta-analytic database of primary studies of these disorders. We then employed multilevel kernel density analysis (MKDA) with Monte-Carlo simulations to correct for multiple comparisons as well as ensemble thresholding to reduce cluster size bias to analyze primary fMRI studies of mood, anxiety, and anxiety-related disorders followed by application of triple subtraction techniques and a second-order analysis to elucidate the disorder-specificity of the previously identified neural features.

**Results:**

We found that participants diagnosed with mood, anxiety, and anxiety-related disorders exhibited statistically significant (p < .05 – 0.0001; FWE-corrected) differences in neural activation relative to healthy controls throughout the cerebral cortex, limbic system, and basal ganglia. In addition, each of these psychiatric disorders exhibited a particular profile of neural features that ranged from disorder-specific, to category-specific, to transdiagnostic.

**Conclusions:**

These findings indicate that psychiatric disorders exhibit a complex profile of neural features that vary in their disorder-specificity and can be detected with large-scale fMRI meta-analytic techniques. This approach has potential to fundamentally transform neuroimaging investigations of clinical disorders by providing a novel procedure for establishing disorder-specificity of observed results, which can be then used to advance our understanding of individual disorders as well as broader nosological issues related to diagnosis and classification of psychiatric disorders.

**Disclosure of Interest:**

None Declared

